# Ampelopsin E Reduces the Invasiveness of the Triple Negative Breast Cancer Cell Line, MDA-MB-231

**DOI:** 10.3390/molecules24142619

**Published:** 2019-07-18

**Authors:** Francis Yew Fu Tieng, Saiful Yazan Latifah, Nur Fariesha Md Hashim, Huzwah Khaza’ai, Norizan Ahmat, Banulata Gopalsamy, Agustono Wibowo

**Affiliations:** 1 Department of Biomedical Sciences, Faculty of Medicine and Health Science, Universiti Putra Malaysia, 43400 Serdang, Selangor, Malaysia; 2 Laboratory of Molecular Biomedicine, Institute of Bioscience, Universiti Putra Malaysia, 43400 Serdang, Selangor, Malaysia; 3 Department of Chemistry, Faculty of Applied Sciences, Universiti Teknologi MARA, 40450 Shah Alam, Selangor, Malaysia

**Keywords:** ampelopsin E, triple negative breast cancer, metastasis, invadopodia

## Abstract

Breast cancer is the most common and the second leading cause of cancer-related deaths in women. It has two distinctive hallmarks: rapid abnormal growth and the ability to invade and metastasize. During metastasis, cancer cells are thought to form actin-rich protrusions, called invadopodia, which degrade the extracellular matrix. Current breast cancer treatments, particularly chemotherapy, comes with adverse effects like immunosuppression, resistance development and secondary tumour formation. Hence, naturally-occurring molecules claimed to be less toxic are being studied as new drug candidates. Ampelopsin E, a natural oligostilbene extracted from *Dryobalanops* species, has exhibited various pharmacological properties, including anticancer and anti-inflammatory activities. However, there is yet no scientific evidence of the effects of ampelopsin E towards metastasis. Scratch assay, transwell migration and invasion assays, invadopodia and gelatin degradation assays, and ELISA were used to determine the effects of ampelopsin E towards the invasiveness of MDA-MB-231 cells. Strikingly in this study, ampelopsin E was able to halt migration, transmigration and invasion in MDA-MB-231 cells by reducing formation of invadopodia and its degradation capability through significant reduction (*p* < 0.05) in expression levels of PDGF, MMP2, MMP9 and MMP14. In conclusion, ampelopsin E reduced the invasiveness of MDA-MB-231 cells and was proven to be a potential alternative in treating TNBC.

## 1. Introduction

Breast cancer is the most common menacing health issue affecting women worldwide [1,2] as well as the second leading cause of cancer-related deaths to date. In 2012, there were approximately 1.7 million women diagnosed with breast cancer among which there were 521,900 deaths [3,4]. Breast cancer occurs mainly in the high age group of women over 55 with ratio of 1 to 10 [5]. According to Global Cancer Statistics 2018, breast cancer accounted for one in every four cancer cases with 2,088,849 new cases and 626,679 deaths [6]. Breast cancer is a genetically and clinically heterogeneous disease due to its distinct biological entities that are associated with specific morphological and immunohistochemical features and clinical behaviours, leading towards differences in treatment response patterns and clinical outcomes [7,8,9]. The traditional classification of breast cancers is based on their histological appearance and biological features like tumor size, lymph node involvement, patient’s age, histological grade and status of hormone receptors: estrogen receptor (ER), progesterone receptor (PR) and human epidermal growth factor receptor-2 (HER-2 or c-erbB2) [10]. Breast cancer which lacks the expression of ER and PR and does not show HER-2 overexpression is termed as triple negative breast cancer (TNBC) [11,12,13,14,15]. 

Generally, 10 to 24% of invasive breast cancers are TNBC [16,17,18]. TNBC is a heterogeneous entity, which includes both high grade invasive ductal carcinomas and other low grade molecular subtypes [19,20,21]. This results in shorter overall survival in terms of prognosis [22,23]. Currently, chemotherapy, which consists of taxanes, ixabepilone, anthracyclines, platinum agents, biologic agents and anti-epidermal growth factor receptor drugs, is the only routine systemic treatment for TNBC patients (both in early and advanced-stages) [24]. However, this comes with many drawbacks such as immunosuppression, development of cancer resistance (multidrug resistance) and secondary tumour formation. The reason chemotherapy is still in use is because of the lack of targeted therapies and poor prognosis of TNBC patients (low survival rate). Thus, there is a need in the discovery of naturally occurring anti-cancer molecules with lower toxicity and reduced adverse effects towards the human body [25,26,27,28]. 

Presently the traditional treatment approaches that are being used for breast cancer treatment are being hindered by a number of impediments, mainly due to toxic effects accompanied by drug resistance. Due to this, various therapies have been propounded for the treatment of cancer, many of which use natural products, including vinca alkaloids, taxanes, podophyllotoxins and antracyclines (doxorubicin) [29,30,31]. These plant-derived products also showed promising synergistic action with many chemotherapeutics and improve their efficacy [32]. Some of these combinations are genistein and doxorubicin, which provide a synergistic effect, equol that increases efficacy of tamoxifen, and pomegranate with ability to enhance tamoxifen-induced inhibition on cell viability [33,34]. Other reasons for the preference for natural products over conventional treatments includes easy availability from the natural environment and usually reduced adverse effects towards healthy human cells [35,36]. Furthermore, various natural compounds extracted from plants are reported as effective anticancer compounds, particularly in the treatment of TNBCs. Some examples of these phytochemicals are polyphenols, bioflavonoids, carotene, vitamins and minerals [37,38]. They are capable of suppressing cell growth, migration and metastasis through targeting aberrant/dysregulated signaling pathways present in TNBCs such as Wnt, Notch, NF-κB, PI3K/Akt/mTOR, MAPK, and Hedgehog pathways [39,40,41,42,43,44]. In other words, these phytochemicals could improve the treatment of TNBC, since TNBC lacks three receptors targeted by current chemotherapy regimens [45]. Natural products are also capable of reversing multidrug resistance (MDR) by regulating drug-resistant proteins [46,47]. For example, flavonoids contain phenylchromane ring with strong affinity for P-glycoprotein (P-gp), which could reverse MDR via inhibiting P-gp transport system [48]. In short, natural products show potential either as alternative or anti-cancer drug candidates in the treatment of TNBC.

**Figure 1:**
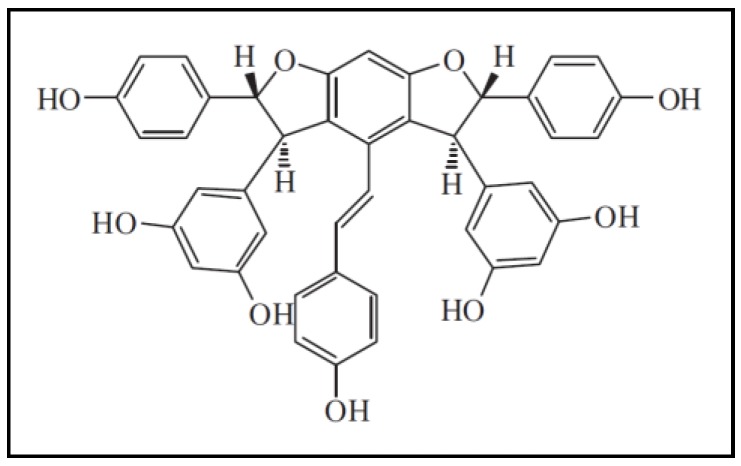
Chemical structure of ampelopsin E, the major active compound isolated from *Dryobalanops*.

In this study, the chosen natural product is *Dryobalanops* (Dipterocarpaceae family), locally called ‘Kapur’ [49,50] that can only be found in the tropical forests of West Malaysia (Sumatra, Peninsular Malaysia and Borneo) [51,52]. *Dryobalanops* is represented by only seven species worldwide: *D. rappa*, *D. aromatica*, *D. lanceolata*, *D. beccarii*, *D. fusca*, *D. keithii* and *D. oblongifolia*. Traditionally, *Dryobalanops* species are used in medicine in the preparation of toothpastes, powders, diaphoretics and antiseptics, and for the treatment of hysteria, and dysmenorrhea [51,53,54]. Approximately 200 oligostilbenoid constituents have been found in the Dipterocarpaceae family since 2014 [55], and they are reported to have antidiabetogenic, anti-angiogenesis, antimicrobial, anticancer, anti-inflammation, antifungal and hepatoprotective activities [56,57,58,59]. One of the major active compounds from *Dryobalanops* species is ampelopsin E (Figure 1) [60]. Ampelopsin E is an oligomeric form of stilbenoid (an oligostilbenoid) with molecular formula of C_42_H_43_O_9_. It belongs to the phenylpropanoid family, which are majorly synthesized in plants from the amino acids phenylalanine and tyrosine, in response to external stimuli [61]. Ampelopsin E has been proven to be cytotoxic towards breast adenocarcinoma cells, MCF-7 [62]. In our previous study, ampelopsin E induced apoptosis and G_2_/M cell cycle arrest in TNBC cells, MDA-MB-231 [63]. Thus, this study aimed to determine the effects of ampelopsin E towards invasiveness of MDA-MB-231 cells.

One defining hallmark of breast cancer is tumor metastasis, which involves cellular migration and invasion. It is a key factor in breast cancer progression and indicates a more advanced stage with poorer prognosis [64]. During metastasis, extracellular matrix (ECM) degradation and remodeling by secreting proteases are coordinated via formation of invadosome, for instance, invadopodia. Invadopodia on breast cancer cells are actin-rich protrusions with ability to localize proteolytic activity in ECM [65,66,67]. The formation of invadopodia also indicates the potential of cancer cells to perform metastasis [65,68,69] as it proves its ability to facilitate the invasive stages of metastasis such as stromal invasion, intravasation, extravasation and colonization of secondary sites [68]. Thus, targeting invadopodia formation could be an effective way of reducing invasiveness of cancer cells. It is believed that ampelopsin E could reduce the invasiveness of breast cancer cells.

## 2. Results

### 2.1. Cytotoxicity of Ampelopsin E

Cell viability of MDA-MB-231 cells treated with ampelopsin E, a major active compound of *Dryobalanops*, was evaluated using MTT assay at five different concentrations (1.88 μM, 3.75 μM, 7.5 μM, 15 μM and 30 μM). 

**Figure 2:**
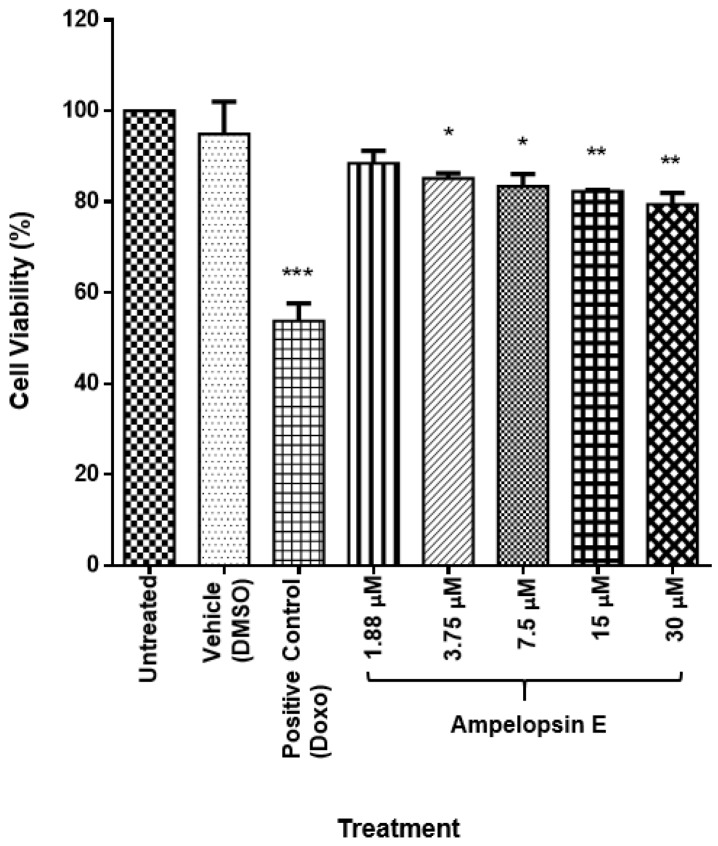
Cell viability of ampelopsin E-treated MDA-MB-231 cells for 24 h. There was a significant reduction in the cell viability of MDA-MB-231 cells at all concentrations of ampelopsin E (3.75 μM, 7.5 μM, 15 μM and 30 μM) following concentration-dependent manner as compared to the untreated group (*p* < 0.05). Results were expressed as mean ± SEM of three independent experiments, *n* = 3. Bar with * indicated *p* < 0.05, bar with ** indicated *p* < 0.01 and bar with *** indicated *p* < 0.001 when compared to untreated group.

There was a significant reduction in the cell viability of MDA-MB-231 cells at all concentrations of ampelopsin E following a concentration-dependent manner as compared to the untreated group (*p* < 0.05) (Figure 2). Comparison was done with untreated group in the entire experiment instead of the vehicle because there was no significant difference between untreated group and vehicle. 

**Figure 3:**
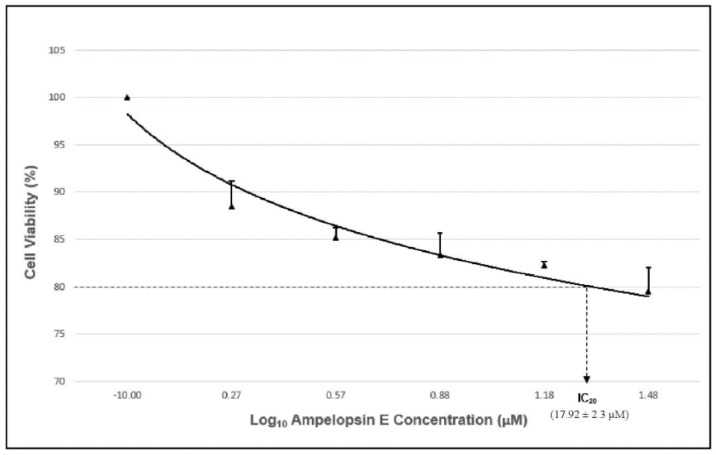
Graph of cell viability of MDA-MB-231 cells against log_10_ ampelopsin E concentration with the IC_20_.

In order to assess the effects of ampelopsin E towards the invasiveness of MDA-MB-231 cells, at least 80% of the cells should be alive to prevent excessive cellular death or apoptosis in the subsequent assays. Since ampelopsin E at a concentration of 30 µM showed a cell viability of less than 80%, it was not incorporated in the entire experiment. The concentration of the compound that caused 20% inhibition of cell growth compared to the untreated group (IC_20_) was obtained from the fit standard curve of percentage cell viability against the concentrations of ampelopsin E. The IC_20_ of ampelopsin E towards the cells at 24-h exposure was achieved at concentration 17.92 ± 2.3 μM (Figure 3). 

### 2.2. Rate of Migration of MDA-MB-231 Cells

A scratch assay was carried out to determine quantitatively and qualitatively the directed migration of MDA-MD-231 cells. Briefly, the monolayer of cells was scratched, and the decrease in the area of scratched cells (cell free area) during the first 24 h upon treatment with ampelopsin E and the rate of migration of MDA-MD-231 cells was assessed. Rate of migration was calculated based on the decrease of cell free area over time using ‘Tscratch’ analysis software.

**Figure 4:**
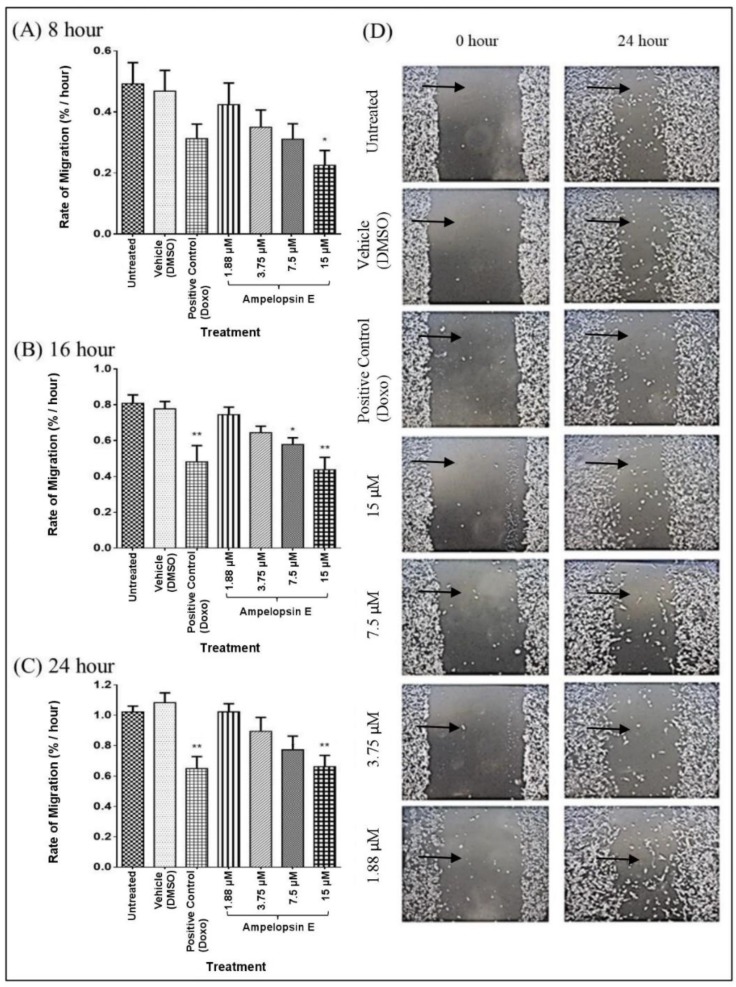
Rate of migration of ampelopsin E-treated MDA-MB-231 cells in DMEM with 10% FBS at 8 h (**A**), 16 h (**B**) and 24 h (**C**). Images were presented at 0 and 24 h at 40× magnification (**D**). Rate of migration was calculated based on the decrease of cell free area (indicated by 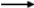) over time using ‘Tscratch’ analysis software. Results were expressed as mean ± SEM of four independent experiments, *n* = 4. Bar with * indicated *p* < 0.05 and bar with ** indicated *p* < 0.01 when compared to untreated group.

Doxorubicin, which was the positive control showed significant decrease (*p* < 0.05) when treated at 16 and 24 h. Any reduction in similar direction signified the ability to reduce cell migration of MDA-MB-231 cells. There was a significant reduction (*p* < 0.05) in the rate of migration of MDA-MD-231 cells (percentage of area/hour) as early as 8 h at 15 μM of ampelopsin E as compared to the untreated group (Figure 4). The most significant (*p* < 0.01) decrease in the rate of migration was observed in cells treated with 15 μM of ampelopsin E at 16 and 24 h when compared to untreated group. Apparent effect of ampelopsin E towards migration of MDA-MB-231 cells was well observed qualitatively with the decrease of the cell free area following increasing concentration of ampelopsin E as early as 24 h. 

**Figure 5:**
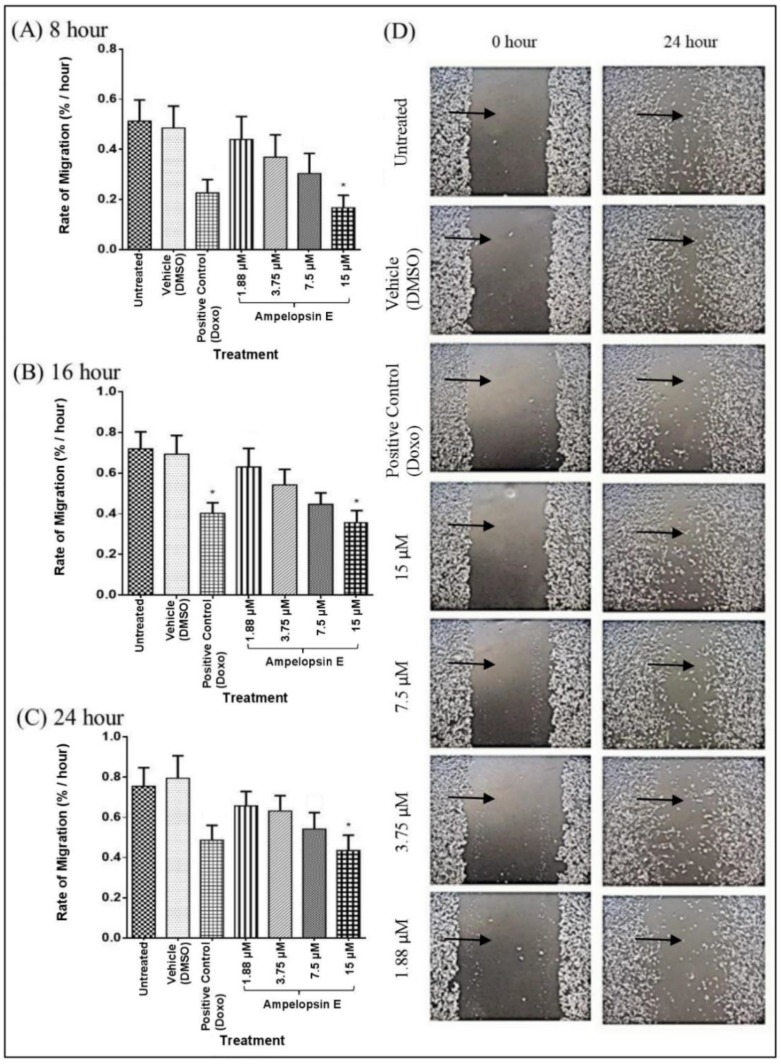
Rate of migration of ampelopsin E-treated MDA-MB-231 cells in DMEM with 2% FBS at 8 h (**A**), 16 h (**B**) and 24 h (**C**). Images were presented at 0 and 24 h at 40× magnification (**D**). Rate of migration was calculated based on the decrease of cell free area (indicated by 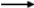) over time using ‘Tscratch’ analysis software. Results were expressed as mean ± SEM of four independent experiments, *n* = 4. Bars with * indicate *p* < 0.05.

In serum-starved condition, there was a significant decrease (*p* < 0.05) in the rate of migration when treated with 15 μM of ampelopsin E at 8, 16 and 24 h (Figure 5) as compared to the untreated group. The reason of performing scratch assay in serum-starved condition was to confirm the strength of previous scratch assay in normal condition.

Serum-starved medium with not more than 2% FBS was only limited to the first 24 h incubation, which was just sufficient to assess cell migration. Prior to the migration assay, the cells were starved overnight. Treatment with ampelopsin E at both normal and serum-starved conditions displayed a similar decline pattern in the rate of migration of MDA-MB-231 cells. This observation was important to proof minimal cellular proliferation in validating the strength of the scratch assay performed in this study. 

### 2.3. Cell Transmigration and Invasion 

Ampelopsin E was shown capable of reducing migration of breast cancer cells. The potential of anti-breast cancer drugs was assessed through inhibition of a multistep process involving migration and metastasis. Therefore, the potency of ampelopsin E in reducing metastasis was assessed through transmigration and invasion of MDA-MB-231 cells.

**Figure 6:**
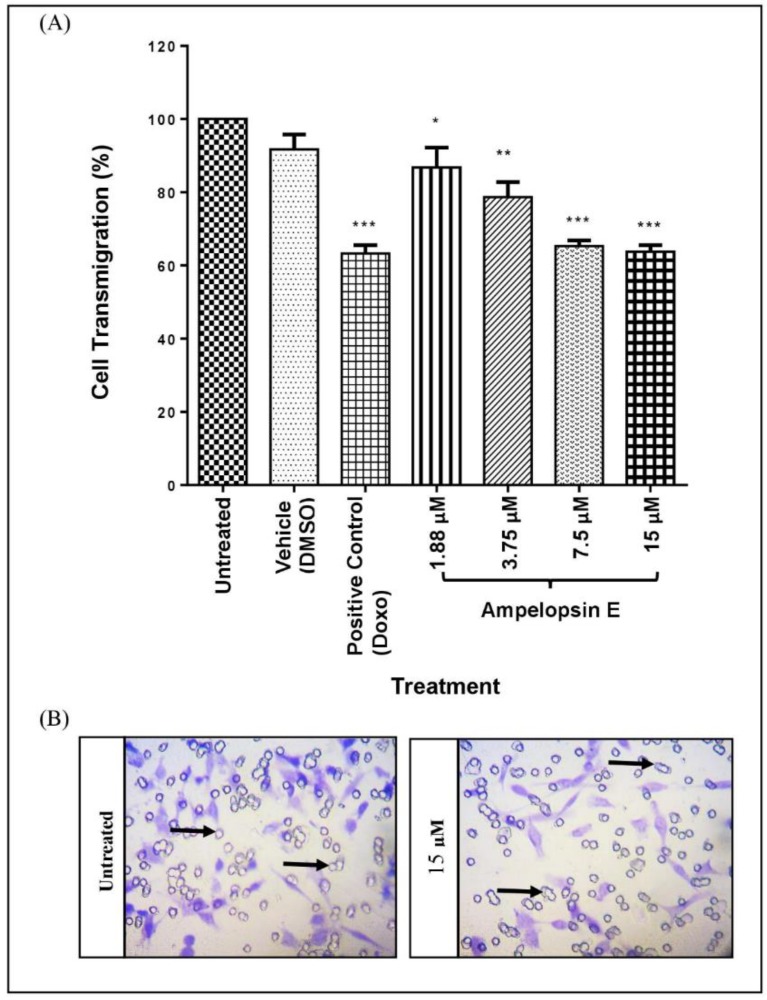
(**A**) Cell transmigration of ampelopsin E-treated-MDA-MB-231 cells normalized to untreated group. Cells were divided into untreated group, vehicle, positive control and four concentrations of ampelopsin E (1.88, 3.75, 7.5 and 15 µM) at 24 h. (**B**) Images were captured at 200× magnification for cell counting. The cells were stained with crystal violet, whereas the hole-like structure in the images were the pores of the transwell membrane (indicated by 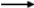). Number of fields = 12. Results were expressed as mean ± SEM of four independent experiments, *n* = 4. Bars with * indicate *p* < 0.05, bars with ** indicate *p* < 0.01 and bars with *** indicate *p* < 0.001 when compared to untreated group.

A transwell migration assay was conducted to determine the ability of MDA-MB-231 cells to perform transmigration via the transwell membrane. Total number of cells entrapped on the membrane was calculated and compared to the untreated group after staining with crystal violet. At 24 h, there was a significant reduction (*p* < 0.05) in cell transmigration following a concentration-dependent manner in all concentrations of ampelopsin E (Figure 6). Treatment at 15 and 30 μM of ampelopsin E demonstrated the highest significance (*p* < 0.001) in the inhibition of cell transmigration of MDA-MB-231 cells comparable to positive control.

**Figure 7:**
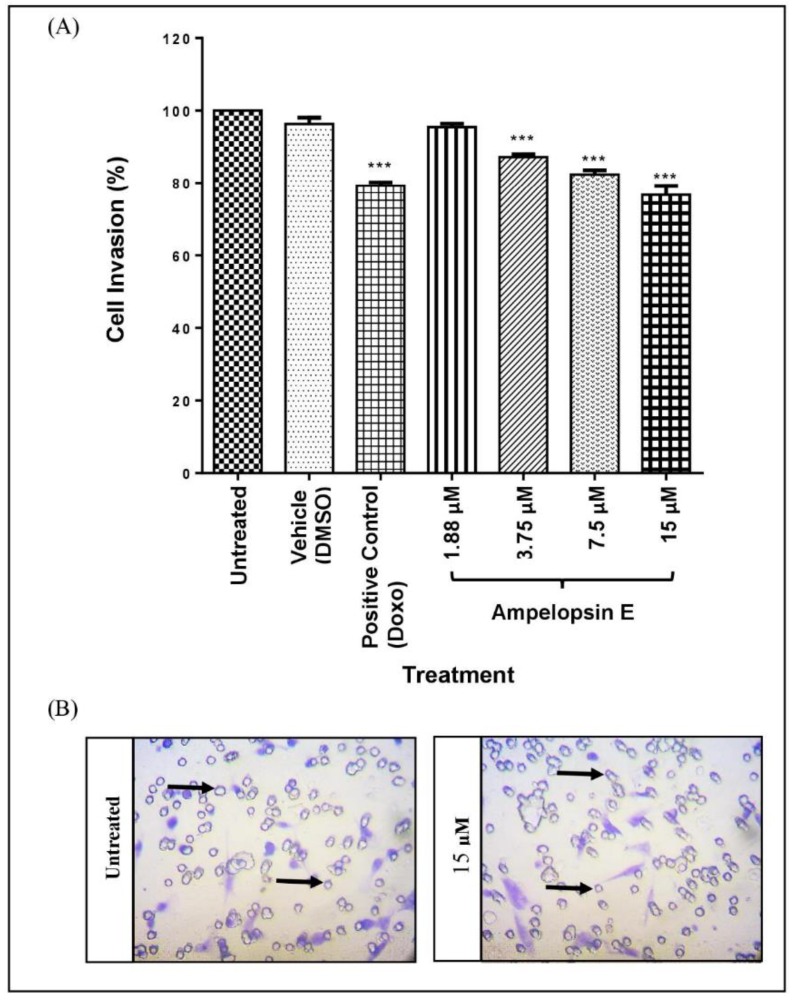
(**A**) Cell invasion of ampelopsin E-treated-MDA-MB-231 cells normalized to untreated group. Cells were divided into untreated group, vehicle, positive control and four concentrations of ampelopsin E (1.88, 3.75, 7.5 and 15 µM) at 24 h. (**B**) Images were captured at 200× magnification for cell counting. Transwell insert membrane was coated with a thin layer of rat tail collagen type I. The cells trapped were fixed and stained with crystal violet, whereas the hole-like structure in the images were the pores of the transwell membrane (indicated by 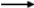). Number of fields = 12. Results were expressed as mean ± SEM of four independent experiments, *n* = 4. Bar with *** indicated *p* < 0.001 when compared to untreated group.

After determining the effects of ampelopsin E towards migration of MDA-MB-231 cells, transwell invasion assay was done to determine the ability of MDA-MB-231 cells to perform invasion. The major difference between the transwell migration and invasion assay was that in the latter, the transwell membrane was coated with a thin layer of rat tail collagen type I. Only the cells trapped within the pores of the membrane were fixed and stained with crystal violet and quantified as result. At 24 h, treatment with ampelopsin E at concentrations above 1.88 μM displayed a significant reduction (*p* < 0.001) in cell invasion as compared to untreated group (Figure 7).

### 2.4. Invadopodia Formation and Gelatin Degradation 

Ampelopsin E has the ability to reduce migration and invasion of MDA-MB-231 cells as demonstrated in the current study. Invadopodia are actin-rich protrusions capable of proteolytic activity. Since invadopodia are known to be a key element in cancer invasion during metastasis, therefore, the correlation between ampelopsin E and invadopodia was assessed.

**Figure 8:**
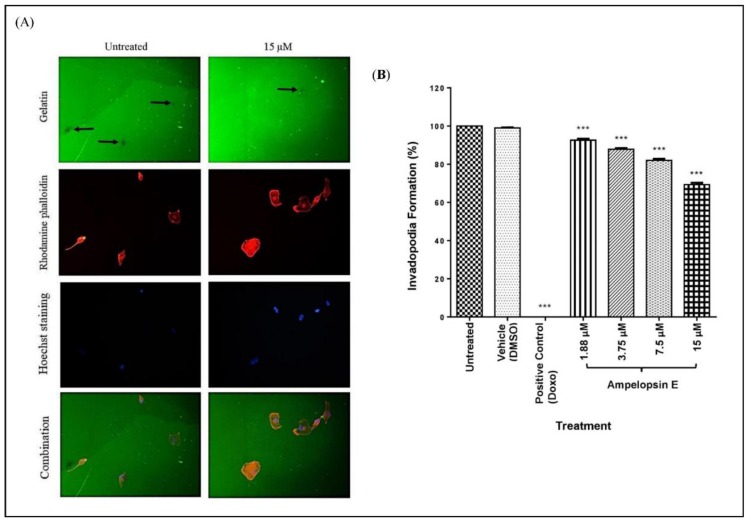
Invadopodia assay. (**A**) Representative images for invadopodia assay. Treated cells were seeded on 488 Oregon Green-gelatin-coated (green colour) coverslips for 3 h, and then fixed and stained with rhodamine phalloidin (red colour) and Hoechst (blue colour). The presence of black dots in the gelatin (indicated by 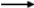) showed that invadopodia had degraded the coated gelatin. Images were captured using a fluorescent light microscope at 200× magnification. Number of fields = 20 and number of cells > 100 per condition. (**B**) Invadopodia formation in MDA-MB-231 cells normalized to untreated group. Cells were divided into untreated group, vehicle, positive control and four concentrations of ampelopsin E (1.88, 3.75, 7.5 and 15 µM) at 24 h. Images were captured using a fluorescent light microscope. Total number of cells with invadopodia and total number of all cells were counted. Number of fields = 20 and number of cells > 100 per condition. Results were expressed as mean ± SEM of four independent experiments, *n* = 4. Bars with *** indicate *p* < 0.001 when compared to untreated group.

Invadopodia assay was carried out to compare the formation of invadopodia across different treatment groups. Briefly, images were captured at 3 different channels with separate colours: 488 Oregon green gelatin (green), rhodamine phalloidin (red) and Hoechst staining (blue). Green colour indicated the layer of gelatin, whereas areas with black dots showed the degradation of gelatin by the MDA-MB-231 cells. Red colour showed the actin filaments of the cells with invadopodia as tiny red dots. Patterns of black dots in gelatin and invadopodia (red dots) were of similar patterns, proving the proteolytic activity of cells on the gelatin layer. The blue staining clearly showed the nuclei of the cells as shown in Figure 8A. 

At 24 h, there was a significant attenuation (*p* < 0.001) in invadopodia formation at all concentrations of ampelopsin E in a concentration-dependent manner as compared to untreated group (Figure 8B). The positive control group (Doxo) successfully inhibit the formation of invadopodia in MDA-MB-231 cells.

**Figure 9:**
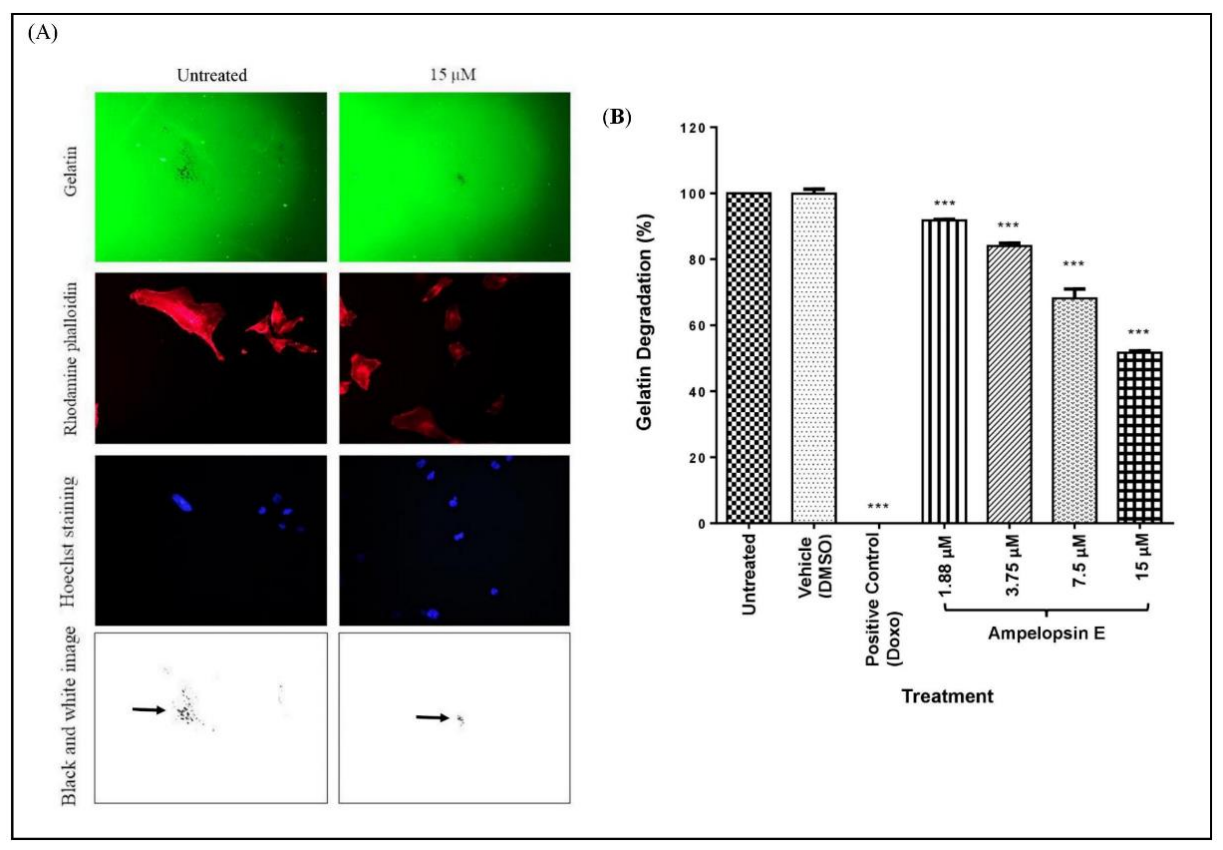
Gelatin degradation assay. (**A**) Representative images for gelatin degradation assay. Images of gelatin degradation were converted into black and white before analyzing using the IMAGEJ software to measure the area fraction (the percentage of degradation area). Black colour/dot (indicated by 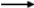) represent the area corresponds to degradation. Number of fields = 20 and number of cells > 100 per condition. Gelatin degradation was calculated by normalizing the area fraction to the number of nuclei. (**B**) Gelatin degradation of MDA-MB-231 cells normalized to untreated group. Cells were divided into untreated group, vehicle, positive control and four concentrations of ampelopsin E (1.88, 3.75, 7.5 and 15 µM) at 24 h. Images were captured using a fluorescent light microscope at 200× magnification. Number of fields = 20 and number of cells > 100 per condition. Area of gelatin degradation was calculated using IMAGEJ analysis software. Results were expressed as mean ± SEM of four independent experiments, *n* = 4. Bars with *** indicate *p* < 0.001 and bars with **** indicate *p* < 0.0001 when compared to untreated group.

Gelatin degradation assay was used to calculate the area fraction (the percentage of degradation area) of invadopodia formed. The images captured previously were converted into black and white. Only the black colour/area was utilized to represent as the area corresponds to gelatin degradation as shown in Figure 9A. Gelatin degradation was calculated by normalizing the area fraction to the number of nuclei of each image. There was a significant decrease (*p* < 0.001) in the gelatin degradation as compared to the untreated group at all concentrations of ampelopsin E at 24 h (Figure 9B). Gelatin degradation of MDA-MB-231 cells were completely inhibited in the treatment of Doxo (positive control).

### 2.5. Proteins Involved in Invadopodia Formation

MDA-MB-231 cells have been proven to perform invasion through gelatin degradation. However, there was no confirmatory tests carried out to validate the formation of invadopodia. Thus, several proteins were chosen to confirm their formation besides giving insights on the way ampelopsin E reduced the invasiveness of MDA-MB-231 cells via invadopodia formation.

**Figure 10:**
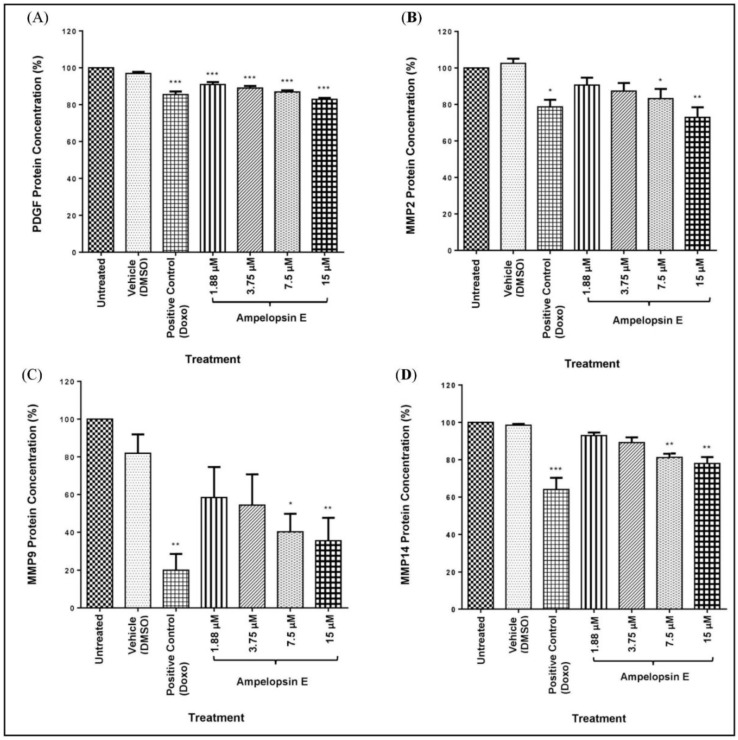
Protein expression of PDGF (**A**), MMP2 (**B**), MMP9 (**C**) and MMP14 (**D**) in ampelopsin E-treated MDA-MB-231 cells normalized to untreated group at 24 h. Protein concentration was quantified via ELISA assay. Results were expressed as mean ± SEM of three independent experiments, *n* = 3. Bars with * indicate *p* < 0.05, bars with ** indicate *p* < 0.01 and bars with *** indicate *p* < 0.001 when compared to untreated group.

Concentration of PDGF was first determined as it was one of the driver in invadopodia initiation. There was a slight decrease (*p* < 0.001) in the expression level of PDGF as compared to the untreated group in all concentrations of ampelopsin E at 24 h as shown in Figure 10A. The highest concentration of ampelopsin E treatment showed similar reduction as the positive control group.

Matrix metalloproteinases (MMP) such as MMP2, MMP9 and MMP14 were highly-expressed during the last step of invadopodia formation (maturation) and usually indicated the formation of functional invadopodia. Figure 10B demonstrates a reduction (*p* < 0.05) of MMP2 at concentration as early as 7.5 μM of ampelopsin E, whereas the most significant decrease (*p* < 0.01) was observed at 15 μM of ampelopsin E as compared to the untreated group. The latter showed promising effects as its value was lower than the positive control group treated with Doxo.

Figure 10C exhibits a dramatic reduction of MMP9 (*p* < 0.05) when treated at 7.5 and 15 μM of ampelopsin E compared to the untreated group at 24 h. Treatment with ampelopsin E showed a marginal reduction (*p* < 0.05) in expression level of MMP14 when treated with 7.5 and 15 μM of ampelopsin E as compared to the untreated group at 24 h (Figure 10D).

## 3. Discussion

Natural products like plants have been used before the discovery of drugs as the primary source of medical treatment [70]. Recently, the use of natural ingredients in the pharmaceutical industry are claimed to be less toxic, hence, reduced adverse effects towards healthy cells [71,72]. Lead compounds derived from plants could also be potential candidates for anticancer treatment [29]. Natural compounds are compounds that are considered as ‘drug-like’ due to their receptor binding capabilities [73,74]. In this study, ampelopsin E is considered as a such a natural compound since it is one of the resveratrol oligomers, which is extracted from *Dryobalanops* [62,75].

According to Wibowo and Ahmat in 2015, ampelopsin E was isolated from *Dryobalanops aromatica* and *Dryobalanops beccarii* using combinations of several chromatography techniques. Briefly, 4 kg of dried stem bark of *Dryobalanops* was macerated with methanol and evaporated under reduced pressure. The dried acetone extract was dissolved in methanol and diethyl ether to produce MeOH-diethyl ether soluble fraction (50.6 g). It was then subjected to refractionation and purification before eluting with EtOAe:n-hexane and EtOAc:MeOH. Four kilograms of *D. aromatica* produced 397 mg of ampelopsin E, whereas 4 kg of *D. beccarii* produced 30.4 mg of ampelopsin E [52,75,76,77,78].

At present, data about the anticancer potential of ampelopsin E is very limited and inconclusive. In 2016, Rahman et al. reported that ampelopsin E had an inhibitory effect on TNBC, by inducing apoptosis and G_2_/M arrest [63]. This approach was realistic, since breast cancer generally involves the rapid and uncontrolled growth of abnormal immortalized cells. Any attempt to halt proliferation (by cell cycle arrest) [79,80] and killing of the cells (by apoptosis) [81,82], resulted in lower proliferation and angiogenesis, hence, prevent cancer from manifesting [83,84]. However, there may be a potential loophole in this scenario, as malignant breast tumor cells are invasive [85]. Invasive breast cancer cells are capable of migration, invasion and metastasis to other body regions, causing breast cancer treatment more intricate [86,87]. Selecting ampelopsin E as an anticancer drug candidate without assessing its invasiveness towards breast cancer cells may result in spreading and relapse of stronger mutated cells. Therefore, this study was designed to evaluate the effects of ampelopsin E towards the invasiveness of MDA-MB-231 cells.

In the previous study, screening of ampelopsin E had been carried out in several cancerous and non-tumorigenic cell lines including MDA-MB-231, MCF-7, HT-29, A-549, HeLa, 3T3 and MCF10A [63,76,78]. Among them, MDA-MB-231 cells shown the lowest IC_50_ value of 14.5 ± 0.71 μM at 72 h, indicating the highest cytotoxicity when treated with ampelopsin E [63]. Thus, MDA-MB-231 was chosen as the target to study the anti-invasiveness capability of ampelopsin E.

Our results showed a percentage of cell viability greater than 80% at concentrations of 1.88, 3.75, 7.5 and 15 µM of ampelopsin E. However, in order to analyse the invasive properties, viable breast cancer cells are required. In this study, IC_20_ value of ampelopsin E at 24 h was determined to prevent excessive cell death or apoptosis which could influence the results of this study. Thus, it is assumed that the four different treatment concentrations of ampelopsin E (1.88, 3.75, 7.5 and 15 µM) are suitable in assessing the invasiveness of MDA-MB-231 cells.

Based on our results, cells treated with ampelopsin E showed a reduction in the rate of migration. Cell migration is an crucial aspect in cancer research, as majority of deaths occurred in cancer patients are related to metastatic progression [64,88]. In order for cancer to spread and disseminate, cancer cells must first migrate and invade into the surrounding host tissues, break extracellular matrix (ECM), intravasate into blood circulation, attach to a distant site, and finally extravasate to form distant foci [83,88,89,90,91,92,93]. Thus, ability of ampelopsin E to inhibit migration of cancer cells may indicate its potential in reducing invasiveness of MDA-MB-231 cells. Furthermore, similar research by Harun et al. in 2018 reported that 2,6-bis-(-4-hydroxy-3-methoxybenzylidine) cyclohexane or BHMC has the ability to prevent breast cancer progression via the inhibition of migration and invasion of MDA-MB-231 cells.

To rule out the effects observed in the migration assay are partly due to the anti-proliferation effects, a scratch assay in serum-starved conditions was performed and it showed similar trends to the previous results. In normal conditions, although the cells were serum-starved overnight to prevent cellular proliferation, the scratch assay was unable to identify the presence and impact of proliferation on cell migration. If proliferation was present, the results obtained may be a false positive data [94]. Interestingly, no proliferation or the proliferation of MDA-MB-231 cells was too minimal to affect the strength of the study. However, there are still some drawbacks of scratch assay that could not be avoided, including the inability to distinguish between the effect of proliferation and changes in cell survival, instability over period of 24 h, differing stain quality, leftover of cells, debris in the scratch area, accidental cell damage, floating apoptotic cells and uneven scratch [95,96]. Even so, as one of the traditional and classical methods, the scratch assay is firmly established as a popular and affordable method in studying cell migration in monolayer culture, while prevailing over other modern approaches such as the cell exclusion zone assay and radius 2D-cell migration assay [97]. Another better option is the use of 3D models like spheroid migration assay and capillary chamber migration assay, where cells can grow naturally and interact with each other, the ECM and microenvironment [98].

After confirming the potential of ampelopsin E in reducing migration capabilities, we then tested its ability in reducing cellular transmigration and invasion of MDA-MB-231 cells. Transwell migration and invasion assays were chosen as they best mimic the process of transmigration, ECM invasion and extravasation by adding a layer of collagen [99]. In metastasis, cancer cells must regain epithelial characteristics in order to anchor themselves in the surrounding host tissues, before penetrating through ECM and then intravasate into the blood vessels, followed by extravasation in order to form secondary tumors at distant sites [100,101,102]. MDA-MB-231 cells were believed to move via mesenchymal type of migration after undergoing epithelial-to-mesenchymal transition (EMT), since they were able to penetrate through collagen-coated membrane [103,104]. This type of migration is characterized by actin-rich invadosome structures such as lamellipodia, filopodia, podosomes and invadopodia, and relies on protease-dependent degradation of the ECM and the formation of strong integrin-dependent contacts for adhesion, migration and invasion of surrounding tissues [105,106,107]. Epithelial-to-mesenchymal transition also plays a crucial role in inducing matrix metalloproteinase production, thereby increasing cell invasion of cancer cells [108,109,110]. Furthermore, according to Liu et al. in 2014, migration and invasion of ovarian cancer cells were proven to be suppressed after incubation with ampelopsin, which indicated the anti-invasiveness activity of ampelopsin [111].

We were able to confirm that MDA-MB-231 cells did undergo EMT, as they were able to form invadopodia which were capable of degrading gelatin and facilitated cell invasion [68,112]. Based on our results, ampelopsin E was found out to be able to suppress invasion through reducing the formation of invadopodia and gelatin degradation ability in MDA-MB-231 cells. Invadopodia are protrusion-like structures, which aided migration of cells and have been proven to be responsible for the motility, invasive and metastatic potential of cancer cells [65,69,113]. They do so by localization of proteolytic activity to areas of cells in contact with ECM, by secreting proteases such as cortactin, fascin, MMPs and PDGF [65,67,114,115,116]. Therefore, suppressing invadopodia formation by ampelopsin E was foreseen to reduce breast cancer metastasis. This is also confirmed by similar studies which prevent metastasis by inhibiting tumor cell invadopodia formation [117,118].

It is interesting to find out that Doxo alone could completely stop the formation of invadopodia and gelatin degradation ability in MDA-MB-231 cells, since Doxo belongs to the anthracycline family which acts during multiple phases of the cell cycle and were considered cell-cycle specific. Briefly, Doxo inhibits topoisomerase II, resulting in DNA double-strand breaks and activates the DNA damage response signalling cascade, guiding recruitment of the repair machinery to these breaks, and failure to do so initiates apoptosis [119,120]. Alternatively, Doxo generates free radicals that leads to lipid peroxidation and membrane damage, DNA damage, oxidative stress, and triggers apoptotic pathways of cell death [121,122]. The first assumption was that Doxo will induce DNA damage [123], resulting in lower number of cells and thus, reducing the formation of invadopodia. The second guess was that Doxo was able to perform invadopodia synchronization, which block invadopodia formation, similar to that of broad-spectrum metalloprotease inhibitor batimastat (BB-94) but may involve different pathways [114]. Furthermore, according to Pichot et al. in 2009, Doxo when used alone, was capable of inhibiting growth, migration and invasion of MDA-MB-231 cells and produce better effect when combine with dasatinib [124]. The third and the most acceptable assumption postulates that Doxo caused cell damage and postpone the formation of invadopodia in MDA-MB-231 cells, since treated cells were given only 3 h to form invadopodia and to assess its degradation properties. Washout of Doxo [125,126] and siRNA knockdown [127] could be done in further study to fully understand and identify the underlying pathways in invadopodia formation of MDA-MB-231 cells.

Ampelopsin E reduced expression level of PGDF in MDA-MB-231 cells. Platelet-derived growth factors consist of a variety of strong mesenchymal cell mitogenic agents and growth chemokines. Their signaling pathway have been extensively studied and well characterized because they regulate many cellular processes, including cell proliferation, migration, invasion, angiogenesis and metastasis [128,129]. They are potent chemoattractants and mitogens, released by tumor cells, for host mesenchymal cells and could mediate the interactions between them for preferred metastatic condition. Furthermore, PDGF is also one of the potential markers for invadopodia initiation [130,131]. Thus, learning the changes of PDGF concentrations in MDA-MB-231 cells gave insights on how the latter response to ampelopsin E. We believed ampelopsin E was able to reduce invasiveness of MDA-MB-231 cells through suppressing PDGF, which promotes EMT via activation of STAT3 or PI3K pathway [132] and are responsible for breast tumor aggressiveness through activation of Notch and NF-κB signaling [128,133].

Based on the results, MMP2, MMP9 and MMP14 expression levels were also reduced in cells treated with ampelopsin E. Production of MMPs correlates with the ability of cancer cells to perform EMT. During breast cancer metastasis, EMT induces MMPs production, which in turn facilitates the process of cell migration and invasion. Previous studies reported that ampelopsin was able to suppress EMT by upregulating genes encoded for cyclooxygenase II which had central role in tumorigenesis via the NF-κB signalling pathway [111,134]. In addition, MMPs are enzymes with proteolytic activity to degrade ECM proteins such as collagen and gelatin [135,136]. Three of the MMPs mentioned previously are proven to be important markers for cancer progression and enriched during invadopodia formation [100,137]. Both MMP2 and MMP9 possess fibronectin repeats which recognize gelatin (denatured collagen) as a substrate [138], whereas MMP14 has the ability to recognize and cleave various ECM substrates including gelatin as well as activate MMP 2 and MMP 9 [139]. 

Combination activity of MMP2, MMP9 and MMP14 is crucial in initiating localized degradation of the gelatin by invadopodia during cancer metastasis [140,141]. Thus, ampelopsin E was believed to reduce MMPs levels that in turn suppress cell migration, invasion and the degradation of gelatin/matrix [142].

Although ampelopsin E had shown promising in vitro anti-invasiveness effects in MDA-MB-231 cells, it does not indicate similar findings in animal or human trials. Optimistically, if the compound was transferred into human setting, oral administration could be the easiest and fastest route of administration. However, as a resveratrol oligomer, ampelopsin E exhibits characteristics such as poor water solubility and fast degradation. On top of that, extensive pre-systemic metabolism of the compound through first-pass glucuronidation and sulphate conjugation in small intestine and liver might affect its efficiency [143,144]. On the other hand, intravenous administration could deliver ampelopsin E in greater amount rapidly and overcome the mechanical constraints of gastrointestinal absorption. Nevertheless, a new formulation would be required due to its nature, and there was no reports of intravenous administration of ampelopsin E in humans throughout the scientific literature [145]. Another possible method of introducing ampelopsin E would be via combination with chemotherapeutic drugs and radiotherapy. For instance, ampelopsin was found to markedly reverse the resistance of K562/ADR cells to doxorubicin, suggesting a synergistic effect [146,147]. Furthermore, ampelopsin was proven to enhance tumor-sensitivity of chemotherapy in hepatocellular carcinoma cells [148]. In short, before transferring ampelopsin E to human setting, more preliminary studies should be carried out.

In summary, this study highlighted the inhibitory effects of ampelopsin E towards the invasiveness of MDA-MB-231 breast carcinoma cells by significantly suppressing migration, invasion, invadopodia formation, gelatin degradation and invasion/invadopodia-related protein expressions such as PDGF, MMP2, MMP9 and MMP14. 

## 4. Materials and Methods

### 4.1. Ampelopsin E

Ampelopsin E was kindly supplied by Department of Chemistry, Faculty of Applied Sciences, Universiti Teknologi MARA (UiTM).

### 4.2. Cell Culture

The independent-hormonal breast adenocarcinoma (MDA-MB-231) cells (ATCC^®^HTB-26™) (Rockville, MD, USA) were maintained in DMEM-high glucose supplemented with 10% FBS and 1% antibiotics (100 IU/mL penicillin and 100 μg/mL streptomycin) at 37 °C in humidified atmosphere of 95% air and 5% CO_2_ incubator (Eppendorf, Hamburg, Germany). The morphology was authenticated using the images obtained from ATCC website. Mycoplasma contamination was tested using Hoechst staining.

### 4.3. Cytotoxicity of Ampelopsin E towards MDA-MB-231 Cells

The MDA-MB-231 cells were cultured in 96-well plates (5.0 × 10^4^ cells/well) for 24 h before treating with ampelopsin E (1.88 to 30 µM) for another 24 h. The cells treated with DMSO served as a vehicle control group, whereas those treated with Doxo acted as the positive control group. The cells without any solvent or treatment served as the untreated control group. For cytotoxicity testing, 20 μL of MTT reagent (5 mg/mL) was added to each well in dark condition before incubating for at least 3 h at 37 °C. Next, the medium was discarded and 100 μL of DMSO was added to dissolve the formazan precipitate in each well. The relative amount of viable cells was determined at 570 nm with reference wavelength 630 nm using a microplate reader (BioTek, Winooski, VT, USA). Cell viability was expressed as a percentage relative to the untreated control, and the IC_20_ (20% inhibition of cell growth compared to the control) was obtained from the fit standard curve of percentage cell viability against the compound concentrations [149]. 

### 4.4. Migration of MDA-MB-231 Cells 

The MDA-MB-231 cells were seeded in 6-well plates and cultured until 90% confluency. Next, serum deprivation (culturing of cells in medium without FBS) was done for 24 h to reduce or abolish proliferation that could confound the evaluation of the cell migratory process. Sterile 200 µL pipette tips were used to produce a scratch with depth of 1 mm on the cell layer for each well, followed by rinsing with PBS wash buffer to remove cellular debris. The cells were then treated with four different concentrations of ampelopsin E (1.88, 3.75, 7.5 and 15 µM) (based on data of cytotoxicity of ampelopsin E towards MDA-MB-231 cells) in two conditions: optimum normal condition (10% FBS) and serum-starved condition (2% FBS). The images were captured at 40× magnification at the same spot for every 8 h after the scratching for 24 h under an inverted light microscope (Olympus, Shinjuku, Tokyo, Japan). An image processing and analysis software “TScratch” was used to determine the percentage of cell free area [89,150]. Rate of migration was calculated based on the percentage of cell free area divided by the duration of treatment by using the following formula:

(1) $$Rate\;of\;migration=\;\frac{Cell\;free\;area\;or\;percentage\;of\;cell\;free\;area}{Duration\;of\;treatment}$$

### 4.5. Transmigration and Invasion of MDA-MB-231 Cells 

Cell culture inserts of 24-well plates (8 μm pore) was used with the top chamber coated and solidified for 2 h with rat tail collagen type I (0.4 mg/mL) for the invasion assay, whereas in the migration assay, the inserts were not coated. Media containing 10% of FBS filled the lower chamber as chemoattractant, whereas in the upper chamber, cells (1.0 × 10^6^ cells/mL) were cultured in media containing 10% of FBS without any solvents or treatment as untreated, media with DMSO as vehicle, media with Doxo as positive control and media with tested concentration of ampelopsin E (1.875, 3.75, 7.5 and 15 μM) for 24 h [151]. After incubation, the non-migrating/invading cells on the upper surface of the membrane were removed using a cotton swab, while the migrated/invaded cells on the lower surface of the membrane were fixed in 70% ethanol for 10 min and left to dry for 10 min. Next, it was stained with 0.2% crystal violet for 10 min at room temperature, followed by washing with distilled water to remove excess staining. Pictures were captured at twelve random fields at 200× magnification under an inverted light microscope (Olympus). Number of migrated/invaded cells was calculated and the results were then converted to percentage and normalized to control (as formula below) [152].

(2) $$\mathrm{Percentage}\mathrm{of}\mathrm{transmigration}\mathrm{or}\mathrm{invasion}\;=\;\frac{\mathrm{Total}\mathrm{number}\mathrm{of}\mathrm{cells}\mathrm{transmigrated}\mathrm{or}\mathrm{invaded}\mathrm{of}\mathrm{treatment}\mathrm{group}}{\mathrm{Total}\mathrm{number}\mathrm{of}\mathrm{cells}\mathrm{transmigrated}\mathrm{or}\mathrm{invaded}\mathrm{of}\mathrm{control}\mathrm{group}}\times 100\%$$

### 4.6. Preparation of Gelatin-Coated Sterile Coverslip

Round coverslips were treated with 70% ethanol for 30 min, 95% ethanol for another 30 min and then left to dry in a drying oven. Next, 488 Oregon Green gelatin (0.2 mg/mL) diluted in 2% sucrose in PBS wash buffer was used to coat the sterile coverslips for 10 min. Next, the coverslips were incubated with 0.5% glutaraldehyde for 15 min to crosslink the gelatin, followed by rinsing with PBS wash buffer twice to remove excess glutaraldehyde. The coverslips were incubated with 5 mg/mL sodium borohydride for 3 min before rinsing with PBS wash buffer for three times. The coverslips were then sterilized with 70% ethanol for 5 min, followed by drying in the biosafety cabinet. All the steps were done in the dark at room temperature. The coverslips were either used on the same day or stored in PBS wash buffer with 2% antibiotics at 4 °C in the dark for up to 2 weeks [153,154].

### 4.7. Invadopodia Detection 

The MDA-MB-231 cells were incubated (1 × 10^5^ cells/mL) in 6-well plates overnight before treatment with ampelopsin E (1.875, 3.75, 7.5 and 15 µM) for 24 h. At 22 h, sterile gelatin-coated coverslips were rinsed with PBS wash buffer for three times before incubation with complete media only for not less than 1 h. At 24 h, the treated MDA-MB-231 cells previously (2 × 10^4^ cells/mL) were seeded onto the coverslips and incubated for 3 h at 37 °C (one coverslip per well in 24-well plates with gelatin-coated surface facing upwards). Next, the media was removed before rinsing with PBS wash buffer once, followed by fixing with 4% paraformaldehyde immediately for 20 min. The cells were then rinsed again with PBS wash buffer for three times before incubating in 0.2% triton X-100/PBS for 5 min. The cells were washed with PBS wash buffer for three times. Next, the cells were incubated with 1% rhodamine phalloidin diluted in 3% bovine serum albumin (BSA) for 1 h, followed by Hoechst staining for 1 min. The cells were washed with PBS twice, followed by distilled water once to remove excess staining. All the steps were done in the dark at room temperature [153,154].

The coverslips were mounted by inverting them over glass slides containing a drop of mounting medium before imaging under a fluorescence microscope (Olympus). Invadopodia were detected in the “red” channel as F-actin rich puncta in the ventral surface of the cell in contact with gelatin, whereas gelatin degradation was detected in the “green” channel as dark areas over the green background. The activity of invadopodia was defined by the average number of punctate gelatin degradation per cell in twelve random fields (>100 cells) [155,156,157]. 

### 4.8. Gelatin Degradation

The images captured were converted into black and white in order to calculate the gelatin degradation by MDA-MB-231 cells. IMAGEJ software was used to measure the area fraction of gelatin degradation (the percent of area that corresponds to degradation). Gelatin degradation was calculated by normalizing the area fraction to the number of nuclei using the formulas below [158]:

(3) $$Gelatin\;degradation=\;\frac{Total\;area\;fraction\;obtained\;from\;ImageJ\;software}{Total\;number\;of\;cells}\;$$

(4) $$Gelatin\;degradation\;\left(percentage\;of\;control\right)=\;\frac{Gelatin\;degradation\;of\;treated\;groups}{Gelatin\;degradation\;of\;control\;}\times 100\%$$

### 4.9. Detection of Matrix Metalloproteases and Platelet-Derived Growth Factor 

The MDA-MB-231 cells were cultured until 80% confluency at 37 °C before treatment with ampelopsin E (1.875, 3.75, 7.5 and 15 µM). After treatment, the old medium was discarded and MDA-MB-231 cells were gently rinsed once with ice cold 1× PBS wash buffer. The cells were then scraped off and transferred to pre-cooled microcentrifuge tubes. For each 1.0 × 10^6^ cells, 150 to 250 µL of pre-cooled 1× PBS wash buffer was added to keep the cells suspended. The cell suspensions were stored overnight at −20 °C and defrost on the next day. After at least 2 freeze-thaw cycles to break the cell membrane, the cell lysates were sonicated for 10 s at 4 °C twice before the centrifuge process (10 min at 1500× *g* at 4 °C). Next, cell lysates (supernatant) were collected. For matrix metalloproteases (MMP2 and MMP9), the culture supernatants from compound-treated cultures were collected and centrifuged to remove debris. The samples were either assayed immediately or aliquoted and stored at −20 °C for not more than 1 month. The concentrations of MMP2, MMP9 and MMP14 and platelet-derived growth factor (PDGF) were measured by enzyme-linked immunosorbent assay (ELISA) according to the instructions on the kits (Elabscience, Houston, TX, USA). The OD values were measured at 450 nm using a microplate reader (BioTek) [159].

### 4.10. Statistical Analysis

Data were expressed as mean ± standard error of mean (mean ± SEM). Data analysis was carried out by Statistical Package for the Social Sciences (SPSS) version 21.0 (International Business Machines (IBM) Corporation, Armonk, NY, USA) through one-way analysis of variance (ANOVA) and Dunnett’s multiple comparisons test. A p value less than 0.05 was considered as statistically significant.

## 5. Conclusions

Ampelopsin E was able to halt migration, transmigration and invasion in MDA-MB-231 cells by reducing formation of invadopodia and its degradation capability through reduction in concentrations of PDGF, MMP2, MMP9 and MMP14. The results of this study showed that ampelopsin E portrayed a great potential as an alternative in treating TNBC.

## 6. Future Directions

The findings of this study suggest a positive impact of ampelopsin E towards invasiveness of MDA-MB-231 cells. However, there are still many unknown mechanisms underlying the anti-breast cancer properties of ampelopsin E, hence in vitro and in vivo models should be established and studied. For example, gene knockout or the use of inhibitor of the respective signaling pathways could be performed. In terms of animal models, anti-breast cancer properties and toxicity of ampelopsin E should be studied. Although ampelopsin E has been proven to have remarkable anti-breast cancer properties in vitro, data from in vivo studies are by far more convincing as they mimic the conditions present in human body. Moreover, more tests regarding safety and efficacy of ampelopsin E should be carried out to determine its cytotoxicity towards living subjects and their metabolisms before transferring to human setting in the future.

## Abbreviations

2D	Two-dimensional
3D	Three-dimensional
ANOVA	Analysis of variance
BHMC	2,6-bis-(-4-Hydroxy-3-methoxybenzylidine) cyclohexane
D.	*Dryobalanops*
DMSO	Dimethyl sulfoxide
Doxo	Doxorubicin
ECM	Extracellular matrix
EMT	Epithelial-to-mesenchymal transition
ER	Estrogen receptor
HER-2 or c-erbB2	Human epidermal growth factor receptor-2
MMP	Matrix metalloprotease
MTT assay	3-(4,5-Dimethylthiazol-2-yl)-2,5-diphenyltetrazolium bromide assay
PDGF	Platelet-derived growth factor
PR	Progesterone receptor
TNBC	Triple negative breast cancer
